# Multimodal chemo-/magneto-/phototaxis of 3G CNT-bots to power fuel cells

**DOI:** 10.1038/s41378-019-0122-x

**Published:** 2020-03-23

**Authors:** Shirsendu Mitra, Nirmal Roy, Surjendu Maity, Dipankar Bandyopadhyay

**Affiliations:** 1 Department of Chemical Engineering, Indian Institute of Technology Guwahati, Guwahati, Assam 781039 India; 2 Centre for Nanotechnology, Indian Institute of Technology Guwahati, Guwahati, Assam 781039 India

**Keywords:** Carbon nanotubes and fullerenes, Nanoparticles

## Abstract

We report the development of a 3G microswimmer, namely, CNT-bot, capable of undergoing acid-, alkali-, magneto- and phototaxis inside acidic or alkaline baths of peroxide fuel and/or water. The use of carboxyl-functionalised multi-walled carbon nanotubes (MWCNTs) facilitated the propulsion of CNT-bots in an alkaline-water solution by ejecting carbon-dioxide bubbles. Furthermore, doping of magnetite nanoparticles (FeONPs), ferrous ions (Fe^2+^) and titanium dioxide nanoparticles (TiONPs) induces magnetic, chemical and photonic modes of propulsion. While FeONPs stimulated magnetotaxis at a rate of up to ~10 body lengths per second under the influence of a bar magnet, chemotaxis of a similar speed in a peroxide fuel was achieved by bubble-propulsion of oxygen gas originating from the Fenton reaction. In addition, the light-stimulated photo-Fenton reaction led to phototaxis of CNT-bots. A thin coating of magnesium imparted a half-faced Janus appearance to the CNT-bots, which facilitated motion in normal or acidic water media through the ejection of hydrogen gas bubbles. This chemotaxis could be transformed into pH-stimulated directional motion by establishing an acid or alkali concentration gradient across the peroxide and/or water baths. The capacity of CNT-bots to produce oxygen (hydrogen) bubbles in peroxide (acidic water) fuel was exploited to power a PEM fuel cell to generate electricity. The pure oxygen and hydrogen gases generated by CNT-bots in separate chambers were fed directly into the fuel cell in which the incessant motions of the particle facilitated the creation and release of the pure gases to achieve on-demand electricity generation. The motor could also induce dye degradation through advanced oxidation owing to the production of intermediate hydroxyl radicals during the Fenton reaction.

## Microrobotics: Multimodal CNT-bots to power fuel cells

Self-propelling objects have been increasingly used for a wide range of applications. Thus far, the first- (1G) and second-generation (2G) motors have served the purpose of achieving diverse motions under a directional trigger. A team headed by Shirsendu Mitra and Dipankar Bandyopadhyay at the Indian Institute of Technology Guwahati, India, has developed a third-generation (3G) artificial microswimmer composed of chemically modified multi-walled carbon nanotubes (MW CNTs)—namely the CNT-bots. Such microbots can move in response to acidic, alkaline, magnetic, and light stimuli. The team used the capacity of the CNT-bots to produce pure oxygen and hydrogen, allowing a proton-exchange membrane (PEM) fuel cell to generate an electric field. The authors believe that their study highlights the potential of a 3G CNT-bot for energy harvesting.

## Introduction

The recent advent of biomimetics of diverse natural products and processes inspires the usage of carbonaceous materials for a host of state-of-the-art applications^1,2^. For example, the synthesis of artificial self-propelling objects for drug delivery^3–5^, environmental remediation^6,7^ or healthcare^8^ applications not only emulates various cellular or subcellular processes^9–14^ but often employs carbon derivatives^15,16^. Importantly, natural processes also motivate the judicious usage of inorganic materials in addition to carbon to achieve improved efficiency and functionality. For example, over the past few decades, a host of organic^17,18^, inorganic^4,19–21^ and composite self-propellers have shown improved propulsion under various excitations, such as photonic excitation^22^, concentration gradients^23,24^, surface tension gradients^25,26^ and electric^27,28^, magnetic^29^ or acoustic^30,31^ fields. In this regard, the existing literature suggests that, while first-generation (1G) motors were synthesised to identify the roles of the materials^32^, size reduction and transport properties^33^ in diverse locomotive behaviour, the major focus during the fabrication of second-generation (2G) locomotives has been functionality, directionality^34^ and biocompatibility^35^. The recent emphasis on the design and development of third-generation (3G) motors is directed towards achieving control over multimodal directional transports suitable for scalable diverse energy^36^, environmental^7,35–38^ and healthcare applications^10,39^.

For example, a number of previous studies have shown the utility of carbon nanotubes (CNTs)^40,41^, graphene^42,43^ and their derivatives as 3G self-propellers, which have been employed as a proof of concept for dye decomposition, drug delivery^44,45^, enzymatic propulsion^46^ and healthcare applications^35,39^. On the other hand, a number of studies have shown the importance of 3G microswimmers for various energy applications. For example, iron (Fe)-based^47,48^ or magnesium (Mg)-based^35^ motors have been shown to have utility in the production of pure hydrogen (H_2_) suitable for fuel cell applications. A few seminal contributions have also demonstrated the capacity of Mg-based motors to decompose hazardous material^49^ and to be used in other in vivo applications^21,49^. It may be noted here that a few previous studies have attempted to use iron nanoparticles to catalyse reactions of organics such as formic^49^ and citric^50^ acids to cause bubble-propulsion of motors via hydrogen production. Subsequently, a number of works have also shown that the hydrogen produced from such sources and the oxygen produced from the decomposition of peroxide fuel using iron nanoparticles can directly be fed into fuel cells for real-time energy harvesting^35,47,48,50^. However, previous work suggests that the use of carbonaceous materials to achieve multimodal self-propulsion in addition to a multitude of other applications has not been attempted thus far. In particular, the alkali-, acid- and phototaxis of such self-propellers via the chemical decomposition of water remains a long-standing challenge.

**Fig. 1:**
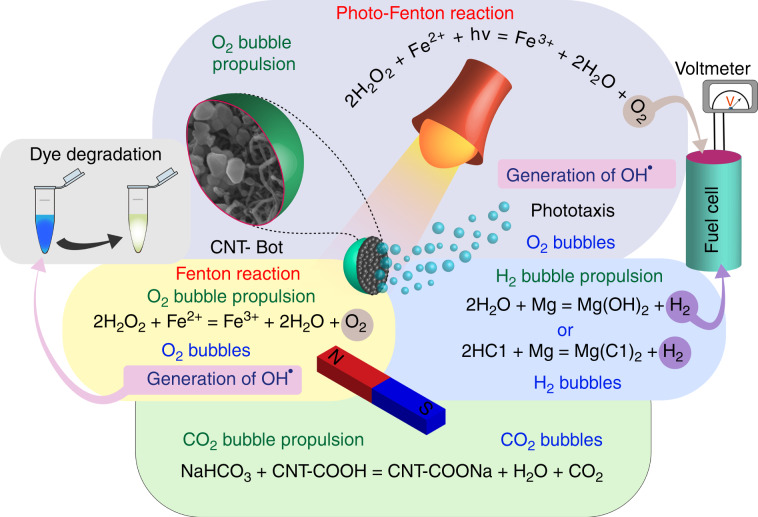
Schematic of a CNT-bot composed of a –COOH-functionalised MWCNT cluster doped with a ferrous (Fe^2+^) salt and magnetite nanoparticles (FeONPs) before being coated with a Mg film. The CNT-bot was capable of undergoing two reactions in acidic and alkaline water (e.g. Mg + H_2_O → Mg(OH)_2_ + H_2_, Mg + HCl → MgCl_2_ + H_2_ and NaHCO_3_ + MWCNT-COOH → MWCNT-COONa + H_2_O + CO_2_) and the Fenton reaction (in the absence of light) and photo-Fenton reaction (in the presence of UV light) in a peroxide fuel (Fe^2+^ + H_2_O_2_ → Fe^3+^ + H_2_O + O_2_), as shown in the image. Subsequently, the motor moved by ejecting hydrogen bubbles in acidic water, carbon-dioxide in alkaline water, and oxygen bubbles in the peroxide fuel. The motors showed directionality in their motion under acid and alkali gradients, leading to acid- and alkali-taxis, as shown in the images. The scheme also shows that hydrogen and oxygen serve as fuels of the PEM fuel cell and that the CNT-bot can also perform a dye degradation function.

In view of this background, we report the design and development of a 3G self-propeller, namely, CNT-bot. Figure 1 shows that in a single embodiment, the 3G CNT-bot shows multimodal directional propulsion in the form of alkali-, acid-, magneto- and phototaxis under chemical, magnetic or photonic stimuli in multiple fluidic media such as water, bicarbonate and hydrogen peroxide solutions. For this purpose, we initially doped –COOH-functionalised multiwall CNTs (MWCNTs) with magnetite nanoparticles (FeONP) and ferrous (Fe^2+^) salt to endow the material with catalytic and magnetic properties. The presence of FeONPs ensures that the motor can undergo magnetotaxis in any fluidic medium in the presence of a magnet, while the presence of Fe^2+^ ensures that the motor can undergo chemotaxis in a peroxide medium via ejection of O_2_ bubbles. Next, a thin layer of Mg was coated on the FeONP-Fe^2+^-doped CNT-bot to exploit the capacity of Mg to decompose acidic water and stimulate acid-taxis through the ejection of H_2_ bubbles. Furthermore, the presence of –COOH functionalisation on the surface empowers the same CNT-bot to eject carbon-dioxide (CO_2_) bubbles in alkaline-water to stimulate alkali-taxis. Interestingly, the CNT-bot can also show directional acid- and alkali-taxis in aqueous and peroxide media when a pH gradient is established. Furthermore, doping of CNTs with TiO_2_ nanoparticles (TiONPs) leads to the formation of photoactive CNT-bots, which exhibit light-driven propulsion by means of the photo-Fenton reaction.

Importantly, the production of pure H_2_ and O_2_ gases from acidic water and alkaline-peroxide media can be directly fed into a PEM fuel cell for real-time power generation. In such a scenario, the incessant self-propulsion of the CNT-bot facilitates the production and release of H_2_ and O_2_ gases owing to the generation of local turbulence. This results in the generation of ~150 mV in the PEM fuel cells using ~100 mg of CNT-bots. Furthermore, the motor can also perform dye degradation via advanced oxidation owing to the production of intermediate hydroxyl radicals during the Fenton reaction. Briefly, the reported phenomena are important from the fundamental point of view owing to the capacity of the carbon-based CNT-bots in showing an unprecedented quintuple mode of locomotion in a single system, but these systems can also serve as energy harvesters, dye degraders and pH sensors.

## Results and discussion

### CNT-bot Locomotion

The details of the synthesis and characterisation of such a self-propeller are discussed in sections S1–S12 of the electronic supporting information (ESI) as well as in the materials and methods sections below. The experiments revealed that, in the same system, the 3G CNT-bot was capable of showing different types of motion in various fuels, such as hydrogen peroxide, acidic or normal water and aqueous sodium bicarbonate solutions. In addition to exhibiting chemotactic behaviour, the motor could show magnetic propulsion owing to the presence of FeONPs in the matrix. In every fuel, the particle underwent a specific reaction, which led to the ejection of gas bubbles, resulting in motion of the motor. For example, the CNT-bot was capable of undergoing two reactions in acidic solution (e.g. Mg + HCl → MgCl_2_ + H_2_) or normal water (e.g. Mg + H_2_O → Mg(OH)_2_ + H_2_), and it could also undergo the following reaction in alkaline water: NaHCO_3_ + MWCNT-COOH → MWCNT-COONa + H_2_O + CO_2_. Furthermore, the CNT-bot could also catalytically decompose peroxide fuel, H_2_O_2_ + Fe^2+^ → Fe^3+^ + H_2_O + O_2_, owing to the presence of Fe^2+^, which is more popularly known as the Fenton reaction.

**Fig. 2:**
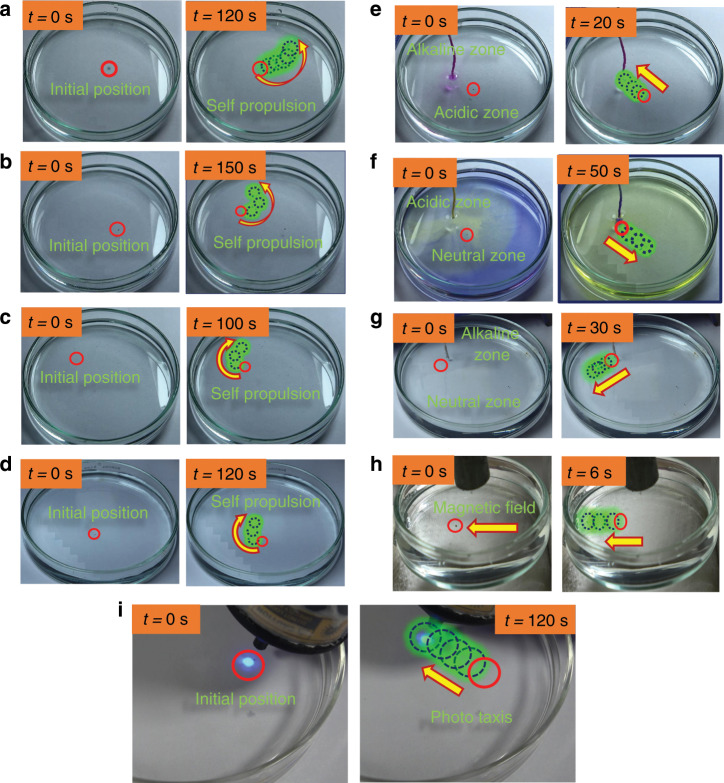
Particle locomotion under various conditions. Image sets **a**–**c** show the trajectory of the random motions of the CNT-bot at different time intervals (labelled on the image) in a hydrogen peroxide bath (12%, v/v), water, and a 0.5 M hydrochloric acid bath, respectively. Image set **d** shows the trajectory of the CNT-bot in a 0.5 M aqueous solution of sodium bicarbonate. Image sets **e**–**g** show the trajectory of the directional motion of the CNT-bot at different time intervals when a concentration gradient was established by dripping acid in a water bath, alkali in a peroxide bath, and bicarbonate in a bath of water. The arrowheads on the images indicate the direction of the movement of the motor. The concentration gradient is indicated by the bromophenol blue, phenolphthalein, and indicators in image sets **e**–**g**, respectively. Image set **h** shows the particle trajectory at different time intervals driven by an externally applied magnetic field. In these experiments, the motor size was ~180 μm to ~300 μm.

To establish these facts, we have shown a detailed GC characterisation of the gases generated during the course of movement in various fuels in Section S2 of the ESI. The chemotactic motions observed for the CNT-bot were largely due to the ejection of H_2_, O_2_, and CO_2_ gas bubbles in acidic water, peroxide fuel and alkaline water, respectively. It may be noted that although the forces due to surface tension or thermal gradients could be present during the experiments, they hardly affect the particle motion, as the bubble propulsion was much stronger than the other kinds of motion. A detailed study on the kinetics of the decomposition reactions of hydrogen peroxide, water, acidic water and alkaline water in the presence of CNT-bots is presented in Sections S3 of the ESI. The diverse propulsions achieved for the CNT-bots are summarised in Fig. 2. It may be noted here that in these experiments, the particle size was kept in the range of ~180 μm to ~300 μm. Image set Fig. 2a and Supporting Video 1 show the random trajectory of the particle moving at an average speed of ~400 µm/s in a 12% (v/v) peroxide solution. Image set Fig. 2b and Supporting Video 2 show the locus of a CNT-bot in a water bath where the speed was found to be somewhat sluggish (~20 μm/s) compared to that in the hydrogen peroxide solution. However, in a 0.5 M HCl solution, the speed was enhanced by three times to ~60 μm/s, as shown in image set Fig. 2c and Supporting Video 3. Furthermore, image set Fig. 2d and Supporting Video 4 show the random motion of the CNT-bot in a sodium bicarbonate bath, where the speed was ~20 μm/s.

Importantly, the motions shown in images Fig. 2a–d were rather random but could be made directional by establishing a concentration gradient in the bath, as indicated by the arrows in image sets Fig. 2e–g. Thus, we dripped aqueous solutions of 1 M HCl, 1 M NaOH, and 0.5 M NaHCO_3_ via cotton threads into the centre of the petri dish, which established the necessary concentration gradient. The dripping via a thread was performed with utmost care to minimise any local convection or disturbances. The images show the presence of the concentration gradient with the help of indicators. Image set Fig. 2e and Supporting Video 5 show the trajectory of the directional motion of the CNT-bot when an external concentration gradient was established by dripping acid into a water bath, as indicated by bromophenol blue. Image set Fig. 2f and Supporting Video 6 show the trajectory of the directional motion of the CNT-bot when an external concentration gradient was established by dripping alkali into a peroxide bath, which was indicated by phenolphthalein. Furthermore, image set Fig. 2g and Supporting Video 7 show directional transport under a NaHCO_3_ drip.

It is well known that alkali acts as a homogeneous catalyst for the decomposition of H_2_O_2_. Thus, dripping alkali at the centre of the peroxide bath created a peroxide-lean zone near the thread and peroxide-rich zones farther from the thread. This was indicated by the colour gradient of the phenolphthalein. In such a scenario, the depletion of peroxide on the surface of the CNT-bot was also non-uniform. For example, less peroxide was depleted from the surface of the CNT-bot closer to the thread than from the surface away from the thread. Subsequently, the difference in the decomposition of peroxide across the surface of the CNT-bot led to directional transport, as shown in image set Fig. 2e. A similar experiment was performed in the water bath when 1 M HCl was dripped from the thread, establishing a low-pH region indicated by the yellow coloration of the bromophenol blue indicator, as shown in image set Fig. 2f. In this situation, a lower (higher) pH near (far from) the thread of the CNT-bot could decompose more (less) water to H_2_. Subsequently, large bubble propulsion on the thread side of the motor led to movement away from the thread, as indicated by the arrow in image set Fig. 2f. Similarly, dripping NaHCO_3_ established an alkali-rich zone near the thread, as indicated by the phenolphthalein indicator in image set Fig. 2g. In this case, a higher (lower) pH near (far from) the thread of the CNT-bot could decompose more (less) NaHCO_3_ to CO_2_. Subsequently, large bubble propulsion on the thread side of the motor led to movement away from the thread, as indicated by the arrow in image set Fig. 2g.

Importantly, the CNT-bots were capable of self-propulsion as well as field-driven motion. For self-population, a chemical bias at the centre of the petri dish was necessary for directional motion, and field-driven motion was mostly directed towards the applied field. For example, image set Fig. 2h and Supporting Video 8 show the magnetic propulsion, which was possible to generate in any of the aforementioned setups. In the case reported, the speed of the CNT-bot was found to be ~2.0 mm/s when the magnetic field was ~155G. In addition to the remotely guided magnetic propulsion, the photoactive CNT-bot loaded with TiONPs could also show light-driven propulsion under the remote guidance of UV light, as shown in image set Fig. 2i and Supporting video 9. Figure 2 and Supporting Videos 1–9 show the diverse propulsive behaviours of the CNT-bot synthesised in a single system.

**Fig. 3:**
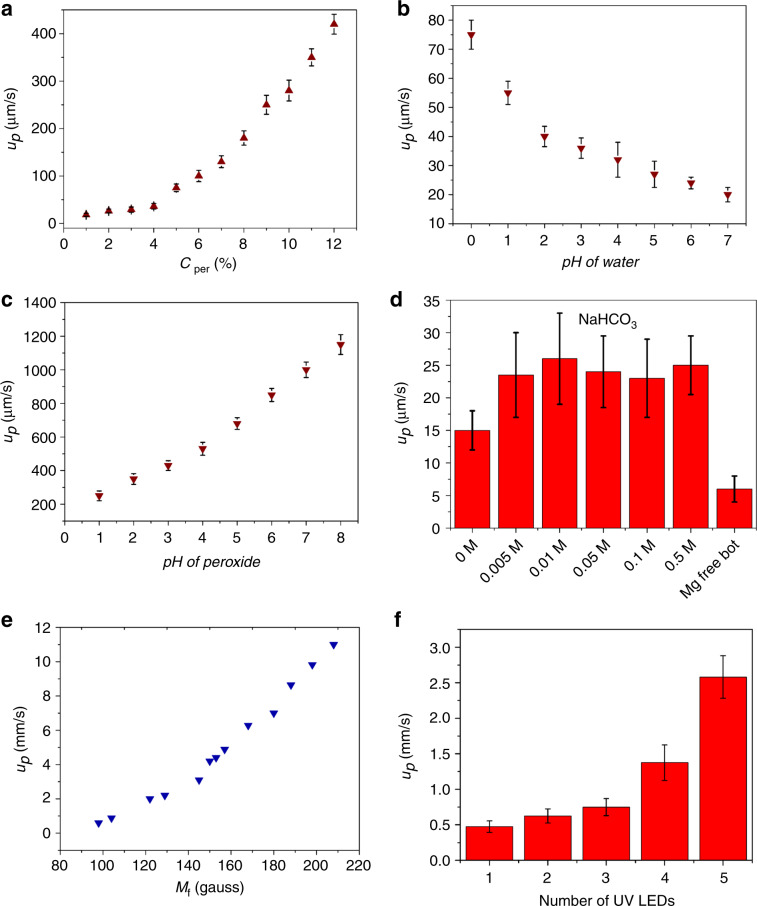
The speeds of CNT-bots with a size of ~180 to 300 μm under different conditions. Plot **a** shows the variation in the speed of the CNT-bot (*u*_*P*_) in an aqueous peroxide bath in which the concentration of peroxide (*C*_*Per*_) was increased from 1% (v/v) to 12% (v/v). Plot **b** shows the variation in *u*_*P*_ when the pH of the water bath was varied. Plot **c** shows the variation in *u*_*P*_ in a 5% (v/v) peroxide bath when the pH of the bath was varied. Plot **d** shows the variation in *u*_*P*_ at different NaHCO_3_ loadings, in pure water, and for a Mg-free swimmer. Plot **e** shows the variation in *u*_*P*_ with different applied magnetic field strengths (*H*) for a 220 μm particle. Plot **f** shows the variation in *u*_*P*_ with the intensity of UV LEDs for photoactive CNT-bots.

A parametric study on the speed of the particles under different stimuli is summarised in Fig. 3. The plot Fig. 3a shows the speeds of ~180–300 μm CNT-bots (*u*_*P*_) in an aqueous peroxide bath. The plot suggests that *u*_*P*_ progressively increased from ~60 µm/s to 400 µm/s with an increase in the concentration of peroxide (*C*_*Pe*r_). Plot Fig. 3b shows that *u*_*P*_ increased from 20 µm/s to 80 µm/s in a water bath when the pH was reduced from 7. Plot (c) shows that in a 5% (v/v) peroxide bath, *u*_*P*_ increased from ~100 µm/s to ~1100 µm/s when the pH of the bath was increased from 1 to 8. We also performed a sensitivity test of *u*_*P*_ in a water bath in addition to a peroxide bath. Interestingly, *u*_*P*_ was found to be higher in the bicarbonate solution than in normal water. A few previous studies have reported that bicarbonate could remove oxide layers from the top of the Mg layer, which may be the reason for this enhancement^35^. To determine the probable mechanism for this phenomenon, we varied the bicarbonate loading in water. Plot Fig. 3d shows that the particle speed increased almost 1.5 times in the bicarbonate solution compared to that in normal water. However, the concentration of bicarbonate had little effect on the particle speed. It may be noted here that the Mg-free CNT-bot reported in this plot is the one with Fe^2+^-doped MWCNTs in which we did not add any magnesium coating for the H_2_ bubble propulsion. The *u*_*P*_ value of this CNT-bot ranged from 3 to 6 μm/s due to CO_2_ bubble propulsion.

The addition of FeONPs in the CNT-bot provided the magnetic handle for the propulsion. Plot Fig. 3e shows the increase in *u*_*P*_ to as high as ~12 mm/s with the increase in the magnetic field strength (*H*) for a ~220 μm particle. In these experiments, the CNT-bot was initially placed inside a petri dish between two poles of an electromagnet before the magnetic field was applied. The field strength was evaluated by a built-in Gauss meter, while the speed was evaluated by image analysis of a recorded video. The magnetic and hysteresis properties of the CNT-bot were also studied with the help of the VSM, which is summarised in Section S4 of the ESI. The photoactive CNT-bots showed a progressive increase in the particle speed with increasing UV light intensity. An experiment was performed with varying light intensity, as shown in plot Fig. 3f. The detailed characterisation of the photoactive CNT-bot is summarised in Sections S5 and S6 of the ESI. Interestingly, the particle speed increased to ~2.5 mm/s in the presence of a UV light source. Here, we assumed that the thermal fluctuations due to UV light and subsequent thermophoresis were rather negligible. Control experiments summarised in Section S7 of ESI also corroborate the correctness of this assumption.

The lifetime of the CNT-bots is another important aspect to be discussed. In peroxide, the CNT-bots could swim for ~180 s without slowing down, whereas the same motor could swim for ~500 s in 0.1 M HCl at the reported speed. In water, the same CNT-bots showed motion for up to ~1000 s before it stopped. The lifetime of the CNT-bots was determined by the kinetics of the reaction, as shown in Section 3 of ESI. We also studied the variations in the speed of CNT-bots with the change in the batch for synthesis, which is summarised in Section S8 of ESI.

### Fuel cell application

**Fig. 4:**
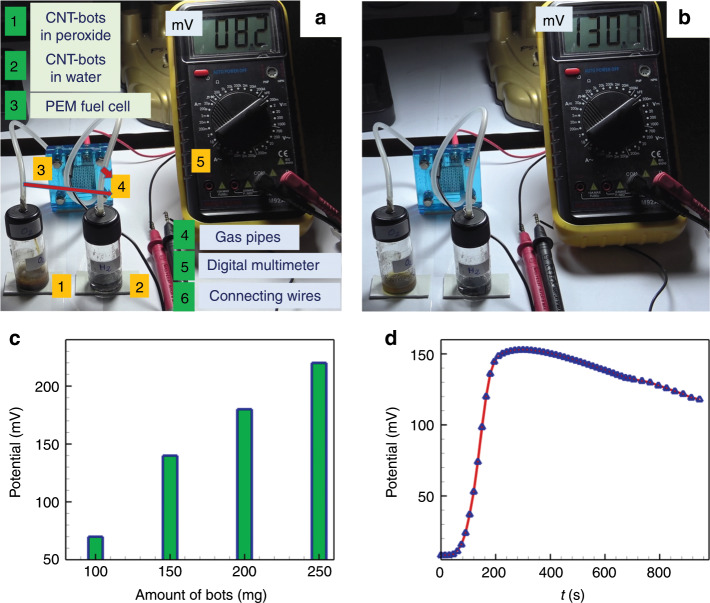
Energy harvesting application of CNT-bots in PEM fuel cell. Image **a** shows the PEM fuel cell setup for electricity generation using the CNT-bots. Containers 1 (filled with CNT-bots and peroxide) and 2 (filled with acidic water and CNT-bots) generated pure O_2_ and H_2_ gases in real time, which were supplied to the fuel cell (3) through gas tubing (4), as shown. The generated current was measured by a digital multimeter (5). Images **a** and **b** show a progressive increase in the potential with time. Image **c** shows the potential developed against the amount of CNT-bots fed in the fuels, and image **d** shows the transient potential output across the PEM fuel cell.

The oxygen gas generated due to the Fenton reaction between the CNT-bots and hydrogen peroxide and the hydrogen gas during the reaction between the CNT-bots and acidic water introduced the opportunity of using CNT-bots in fuel cells to produce real-time electrical energy. Figure 4 and Supporting Video 10 show the setup consisting of a PEM fuel cell in which the electricity was generated with the help of the CNT-bots. In container 1, 50 mg of CNT-bots were added to 10% (v/v) peroxide fuel to generate O_2_ gas, while container 2 was filled with 0.1 M aqueous HCl along with 50 mg of CNT-bots to produce H_2_ gas. The gases thus generated were fed into the PEM fuel cell (3) through gas tubing (4), as shown. The generated current was measured by a digital multimeter (5). The video and images show a progressive increase in the electric field potential with time to up to a value of ~150 mV. The reaction starts with the generation of gas as soon as the CNT-bots are placed in the fuel. Since these reservoirs were connected to a PEM fuel cell by a pipe, the transport of gases through these connectors took some time. In this situation, there are two processes, which happened simultaneously: (i) generation of gases inside the reaction chambers before accumulation of the same in the PEM fuel cell and (ii) consumption and permeation of the ions formed by those gases in the PEM membrane with the anodic (*H*_2_ → 2*H*^+^+2*e*) and cathodic (1/2*O*_2_ + 2*H*^+^ + 2*e* → *H*_2_*O*) reactions^50^. In the initial phase, the rates of generation of gases in the reaction chambers and their subsequent accumulation in the PEM fuel cell were higher than the rate of consumption. Subsequently, the potential increased with time, as shown in Fig. 4d. After some time, the concentration of gases decreased due to the reaction, as shown by the rate kinetics in Section S3 of ESI.

Furthermore, the incessant movement of the CNT-bots in the container facilitated the mixing and de-gassing of the liquids, which helped to generate more voltage with time. At any instance, the partial pressure of the product gases and the consumption of ions in the PEM fuel cell determine the potential across the fuel cell. The role of CNT-bots in increasing the efficiency of the fuel cell is summarised in Section S9 of ESI. In these experiments, equal amounts of magnesium ribbon, magnesium turnings and magnesium in CNT-bots were taken for hydrogen gas generation. The results suggest that the magnesium present in the CNT-bot produced the maximum voltage in the fuel cell.

Supporting Video 11 shows some glimpses of the movements of the motor and the generation and release of gases from the liquid fuel. The details of the electrochemical characterisation of the CNT-bots through cyclic voltammetry (CV) study are summarised in Section S10 of the ESI, clearly showing an ~1.5-fold increase in the current when the working electrodes were decorated with the CNT-bots. Figure 4c shows a significant increase in the electric field potential across the PEM fuel cell with an increase in the amount of motor fed in the reservoirs containing hydrogen peroxide and acidic water as fuels. The experiments revealed that when ~100 mg of CNT-bots were fed into each of the reservoirs, containing ~10% hydrogen peroxide and 0.1 M HCl, a potential output of ~150 mV was generated. In these experiments, the thickness of the magnesium layer on the CNT-bots was ~1 µm, and the average size of the bots was ~180–300 µm. The magnesium content of the motor was ~ 1–2%. Thus, with the increase in the amount of CNT-bots, increases in the production of pure hydrogen and oxygen led to the eventual increase in the potential. However, for a given loading of CNT-bots in the reservoirs, the electric field potential across the fuel cell varied with time, as shown in Fig. 4d. The plot shows that the potential reached a maximum in a short time (~200 s) before starting to decrease at a much slower rate.

### Dye degradation application

**Fig. 5:**
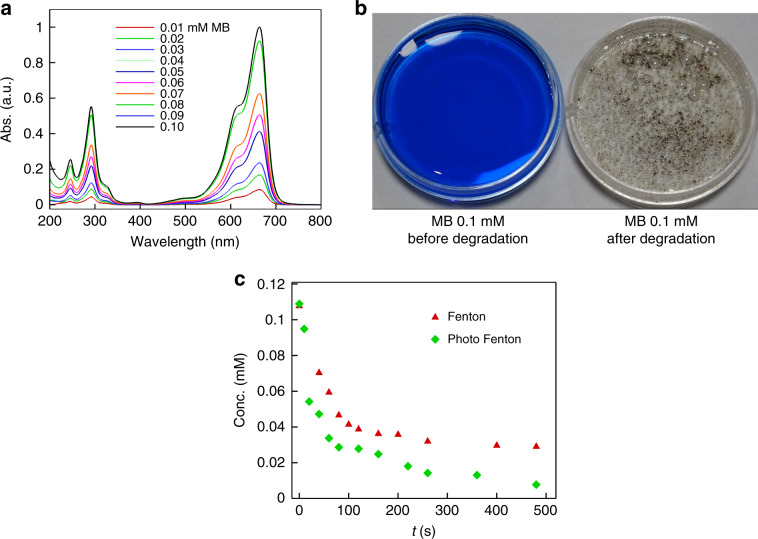
Dye degradation by CNT-bots. Plot **a** shows the UV-Vis absorption spectra of methylene blue solutions of different known concentrations, from 0.01 mM to 0.10 mM. Image **b** shows the colour of a 0.1 mM methylene blue solution before and after degradation using a CNT-bot. Image **c** shows the decay of the methylene blue solution in peroxide fuel in the presence of CNT-bots (red triangular symbols) and photoactive CNT-bots (green diamond symbols).

The CNT-bots were also capable of producing hydroxyl radicals by the Fenton reaction during the course of their motion, and these radicals could also be used for dye degradation and wastewater treatment. To prove this point, 6 ml of ~50% hydrogen peroxide was mixed with 30 ml of a 0.1 mM methylene blue (MB) solution in a petri plate before adding 20 mg of CNT-bots. The experiments initially revealed self-propulsion of the CNT-bots before the blue solution progressively became colourless due to the advanced oxidation reaction by the hydroxyl radicals, as shown in Supporting Video 12. To study the dye degradation kinetics, a set of MB solutions with known concentrations in the range of 0.01–0.1 mM were prepared, and their UV-Vis spectra at 675 nm were obtained, as shown in Fig. 5a. Figure 5b shows the colour of the methylene blue dye in the same petri plate before and after degradation by CNT-bots. Next, the decay kinetics of 30 ml of 0.1 mM MB in 50% H_2_O_2_ and 20 mg CNT-bots were studied. For this purpose, a known amount of sample was collected from this bath every 20 s for a time span of 500 s. The moment CNT-bots were poured into the bath was considered to be 0 s, and the UV-Vis spectra of the pipetted samples were obtained. Figure 5c shows the decay of the MB solution in peroxide fuel in the presence of CNT-bots (red triangular symbols) and photoactive CNT-bots (green diamond symbols). The photoactive CNT-bots showed faster degradation because of the possibility of an additional photo-Fenton reaction, which had higher rate kinetics than the ordinary Fenton reaction.

## Experimental section

### Characterisation

**Fig. 6:**
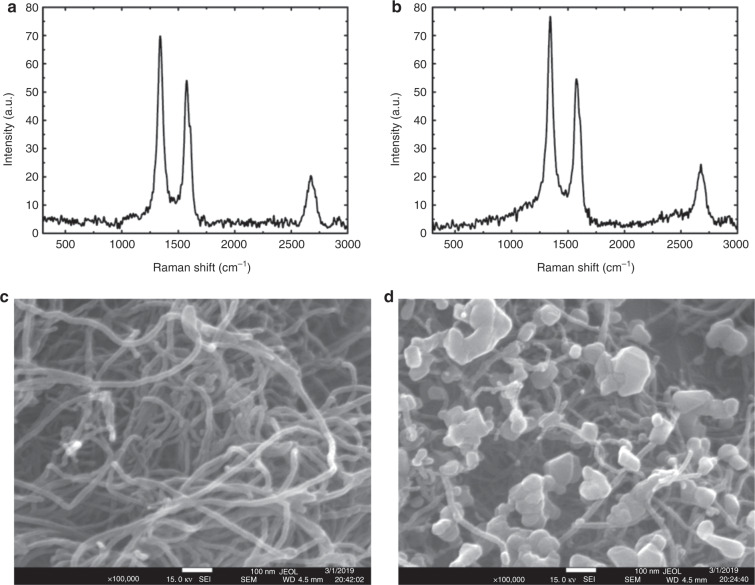
Raman spectra and FESEM images of CNT-bots. Images **a** and **c** show the Raman spectra and FESEM images of pristine –COOH-substituted MWCNTs. Images **b** and **d** show the same Fe^2+^-doped –COOH-substituted MWCNTs.

The ferrous ions doped on the CNT-bot were the major reason for the motion of the particles in hydrogen peroxide. To confirm the doping, we performed Raman spectroscopy and FESEM characterisation to follow the difference before and after doping. Images Fig. 6a, c show the Raman spectra and FESEM images of pristine –COOH-functionalised MWCNTs. Images Fig. 6b, d show the Raman spectra and FESEM images of Fe^2+^-doped –COOH-substituted MWCNTs. The Raman plots show D (1332 cm^−1^) and G (1594 cm^−1^) bands, which were attributed to disorder in and vibration of sp^2^ carbon atoms. The plots reveal that the ratio of I_D_/I_G_ of the Raman spectra increased from 1.290 to 1.401 due to doping of the MWCNT, which could be because of the loading of the Fe^2+^ ions on the MWCNT matrix. This observation was further verified by the FESEM images Fig. 6c, d before and after doping of the MWCNT matrix. Image Fig. 6d clearly shows the presence of nanocrystals of ferrous sulphate on the MWCNTs. The spot EDXS plots of FESEM shown in Section S8 of the ESI also confirmed the presence of –COOH-functionalised MWCNTs and their doping with magnetite and ferrous sulphate in the CNT-bot.

**Fig. 7:**
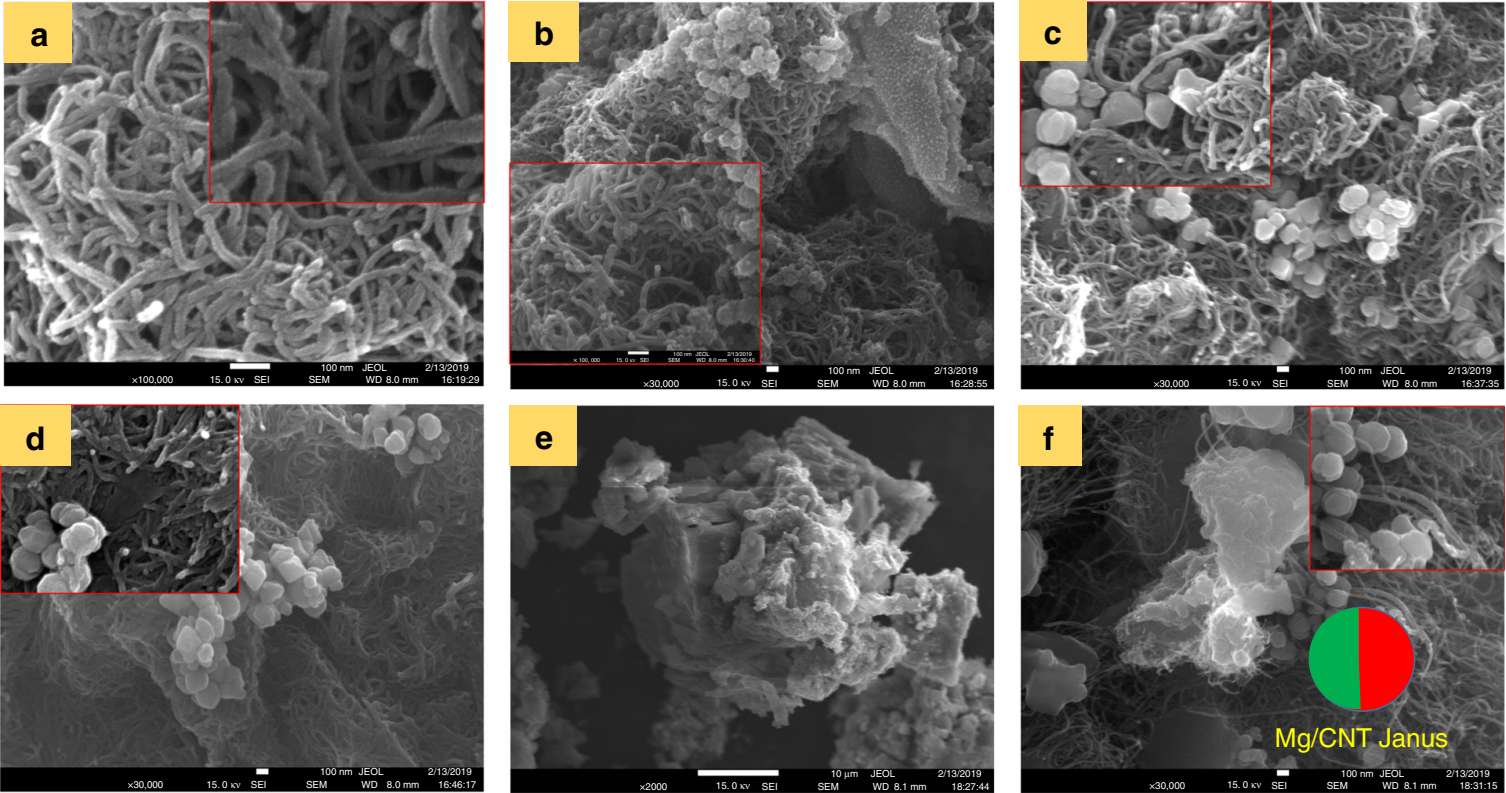
FESEM images of CNT-bots at different stages. Image **a** shows the pristine –COOH-functionalsed MWCNT at ×100,000 magnification, and the inset shows the same image at a higher magnification. Image **b** shows the Fe^2+^-doped MWCNT at ×30,000 magnification, and the inset shows the same at ×10^5^ magnification. Image **c** shows the FeONP-doped MWCNT at ×30,000 k magnification, and the inset shows the same at ×10^5^ × magnification. Image (**d**) shows the FeONP- and Fe^2+^-doped MWCNTs at 30000 × magnification, and the inset shows the same at 10^5^ magnification. Images **e** and **f** show, at lower magnification, the morphology of the Janus-CNT-bot in which one side is FeONP/Fe^2+^-doped MWCNTs, and the other side is magnesium-coated FeONP/Fe^2+^-doped MWCNTs.

Images Fig. 7a, f show the FESEM images of the CNT-bot at the different stages of synthesis. Image Fig. 7a shows the pristine –COOH-functionalised MWCNTs, image Fig. 7b shows the Fe^2+^-doped MWCNTs, image Fig. 7c shows the FeONP-doped MWCNTs and image Fig. 7d shows the composite FeONP-Fe^2+^-doped MWCNTs. Images Fig. 7b, c suggest that ferrous sulphate and magnetite were able to individually penetrate into the MWCNT matrix (image (Fig. 7a)) due to electrostatic interactions. Similarly, image Fig. 7d shows the morphology of the composite of FeONP-Fe^2+^-doped MWCNTs. Images Fig. 7e, f show the surface morphology of the CNT-bot, which suggests that the morphology of the motor somewhat resembled those of the previously reported Janus motors, as schematically shown in the image. In this case, one side of the Janus CNT-bot could be imagined as the red part of the FeONP-Fe^2+^-doped MWCNT, while the green side was magnesium-coated FeONP-Fe^2+^-doped MWCNTs, as schematically depicted in the image. The brightest portions in the images represented magnesium, while the portions with higher contrast represented the MWCNT composites. The spot EDXS plots of FESEM shown in **Section S11** of the **ESI** corroborate the claims made. Further, **Section S12** of the **ESI** shows the XRD study of FeONPs, MWCNT composites, and CNT-bots, confirming the presence of different metals at various stages of fabrication.

## Materials and methods

### Materials

Carboxylic group-substituted multi-walled carbon nanotubes (MWCNT, purity 98%, diameter = 6–13 nm, length = 2.5–20 μm), magnetite nanoparticles (size 50–100 nm), ferrous sulphate, magnesium ribbon, hydrogen peroxide (50% v/v), sodium bicarbonate, hydrochloric acid (33% v/v), sodium hydroxide, phenolphthalein, bromothymol blue, potassium iodide, sulphuric acid and sodium fluoride were procured from Sigma Aldrich, India. The chemicals were of analytical grade and were used without further purification. Millipore water (resistivity, 18.2 MΩ cm; TOC < 5 ppb) was employed to prepare the solutions and wash the synthesised products.

### Methods

Approximately 2 mg of carboxylic acid (-COOH)-substituted MWCNTs and 2 mg of magnetite nanoparticles (FeONPs) were mixed with 1 ml of 0.05 M ferrous sulphate (FeSO_4_) solution before sonication for 30 min to make a colloidal suspension. The resulting suspension was dispensed on a glass slide with the help of a micropipette, and then the slide was gently warmed to evaporate the water from the suspension. In the process, MWCNTs were doped with FeONP/Fe^2+^, and the experimental conditions ensured that the interactions between the FeONP/Fe^2+^ and MWCNT were largely physical. Following this, the glass substrate coated with islands of FeONP/Fe^2+^-doped MWCNTs was coated with magnesium using a thermal evaporator (Make: HHV, India; Model: Lab Coater Auto 500). The deposition was performed five times to coat a thin layer (~750 nm) of Mg on one side of the FeONP/Fe^2+^-doped MWCNTs. Each time, the thermal evaporator deposited an ~150 nm thick Mg layer, which eventually led to a thickness of ~750 nm after five stages of coatings. The thickness of the films was measured using a quartz crystal thickness monitor integrated with a thermal evaporator. The resulting 3G CNT-bots were preserved in an air-tight vacuum desiccator. During the experiments, the CNT-bots were lifted by scraping the glass slide surfaces with the help of a sharp needle. Subsequently, the freshly prepared motors were employed to perform the experiments reported in the present study.

For the photoactive CNT-bots, 15 mg CNT and 30 mg TiO_2_ were initially sonicated in 2.5 ml of ~0.05 M FeSO_4_. The sonicated material was then dried at ~60 °C on a pre-cleaned glass slide. After that, Mg was deposited on top of this material using a thermal evaporator. Each deposition in the thermal evaporator results in a thickness of ~200 nm, and hence, the total thickness of the deposited magnesium layer became ~1 µm after five repetitions. This deposited material was scraped out with the help of a sharp needle before using the materials as microswimmers. The details of the syntheses and characterisation of such self-propellers are discussed in sections S1–S12 of the ESI.

The instruments used to carry out different fabrication and characterisation studies include a thermal evaporator (Make: HHV, India; Model: Lab Coater Auto 500), a video recorder (Make: Sony, Model: FDR AX40), a gas chromatograph (Make: Agilent Model: 7890 A & Make: Thermo Fisher Scientific Trace, Model: 1110), a PEM fuel cell (Vendor: Fuel Cell Store, Product Code: 632000), a field-emission scanning electron microscope (FESEM, Make: JEOL Model: JSM-7610F), an energy dispersive X-ray spectrometer (EDXS, Make: GEMINI 300), an XRD instrument (JCPDS 06-0696), a Raman spectrometer (Make: JEOL, Model: CPX100), a vibrating sample magnetometer (VSM, Make: JEOL, Model: JES-FA200), a UV-Vis spectrophotometer (Make: PerkinElmer, Model: Lambda 35), an electromagnet (Make: SES Instruments, Model: DPS 50), a Gauss meter (Make: SES Instruments, Model: DGM 102), a pH meter (Make: HANNA, Model: EDGE pH), a potentiostat (Make: GAMRY, Model: Reference 6000 + ) and a digital multimeter (Make: MASTECH, Model: M92A (H)).

## Discussion and conclusions

We report the design and development of a 3G CNT-bot capable of four different types of acid- and alkali-taxis in peroxide and water baths in a single system. The movement of the motor could also be remotely controlled with the help of a magnetic field. The Janus type CNT-bots were fabricated by doping magnetite and ferrous ions onto the carboxyl-functionalised MWCNTs before coating one side of them with a thin magnesium layer. While the Fenton reaction facilitated the motion of the CNT-bot in acidic and alkaline-peroxide media through the ejection of oxygen bubbles, the motor could decompose normal or acidic water to produce hydrogen gas for bubble propulsion. The motor could also exhibit carbon-dioxide bubble propulsion in an alkaline bicarbonate solution. The motor movement could be enhanced by increasing the concentration and pH of the peroxide fuel, whereas the speed of the motor increased in the water bath upon reducing the pH. The rate of chemotaxis in the peroxide medium was found to be as high as 10 body lengths per second at an optimal pH and peroxide loading, while the rate was found to be ~1 body length per second in water at a very low pH. The movement of the motors could be remotely guided at a speed of up to ~10 body length per second with the help of a bar magnet. After establishing a gradient of alkali in the peroxide bath, acid in the water bath, and alkali in the water bath, the movement of the motors could be directed. In the process, the CNT-bots showed directional acid- and alkali-taxis emulating the movements involved in cellular and subcellular processes^20–26^. A proof-of-concept prototype for real-time energy harvesting has been demonstrated in which CNT-bots have been employed to generate pure oxygen and hydrogen gases for a PEM fuel cell to generate an electric field potential as high as ~150 mV. The self-propelling CNT-bots facilitated the mixing and de-gassing of the liquids, suitable for real-time voltage generation, as demonstrated. This study highlights the potential of multimodal carbon-based 3G CNT-bots for energy harvesting when functionalised with a small amount of inorganic materials.

### Associated content

Supporting files are also provided along with this article. A doc file containing the descriptions of the details of the characterisation, supporting videos, and nine electronic videos, namely, ‘Supporting Video 1’ to ‘Supporting Video 12’, are available in the electronic supporting information (ESI).

## Supplementary information

Electronic Supporting Informatiom Unmarked

Supporting Video 1

Supporting Video 2

Supporting Video 3

Supporting Video 4

Supporting Video 5

Supporting Video 6

Supporting Video 7

Supporting Video 8

Supporting Video 9

Supporting Video 10

Supporting Video 11

Supporting Video 12
