# Supplementary material for: Multimodal chemo-/magneto-/phototaxis of 3G CNT-bots to power fuel cells
Source: Microsyst Nanoeng. 2020 Mar 23;6:19. doi: 10.1038/s41378-019-0122-x (PMC8433207; doi:10.1038/s41378-019-0122-x)
Supplement: Supplementary file 1 — Electronic Supporting Informatiom Unmarked [file 41378_2019_122_MOESM1_ESM.docx]

**Electronic Supporting Information**

**Multimodal Chemo-Magneto-Photo Taxes of 3G CNT-bots to Power Fuel Cells**

Shirsendu Mitra,^a^ Nirmal Roy,^b^ , Surjendu Maity^b^, Dipankar Bandyopadhyay^a,b*^

*^a^Department of Chemical Engineering, Indian Institute of Technology Guwahati, Assam, 781039, India.*

*^b^Centre for Nanotechnology, Indian Institute of Technology Guwahati, Assam, 781039, India.*

**^*^**Corresponding Author’s Email: [dipban@iitg.ac.in](mailto:dipban@iitg.ac.in)

**S1: Synthesis of CNT-bots**


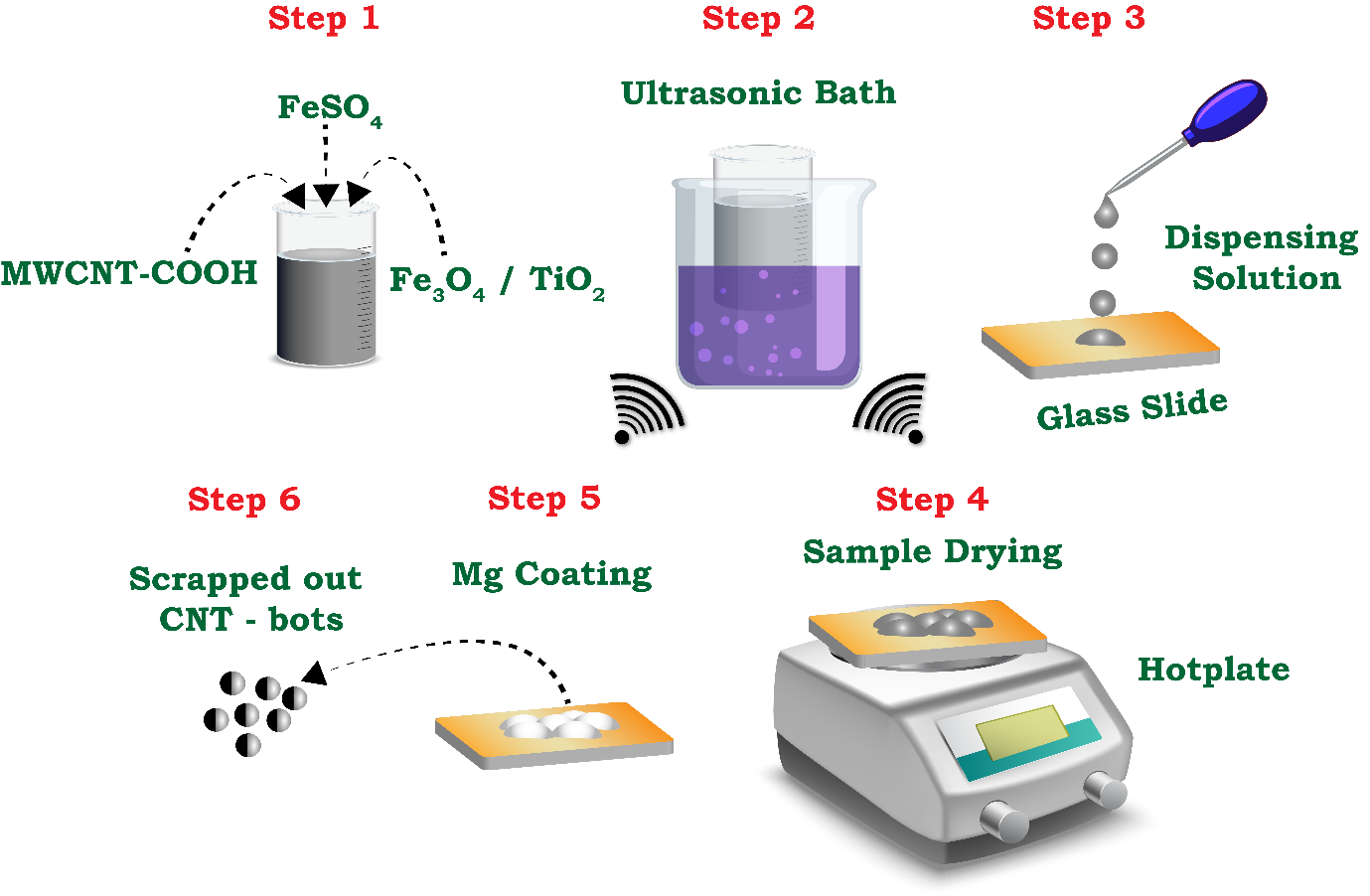


**Figure S1:** Schematically shows different steps of CNT-bot fabrication.

**Figure S1** shows the detailed steps for the fabrication of the CNT-bots. Initially, in the step 1, 15 mg of MWCNT-COOH was mixed with 15 mg of Fe_3_O_4_ nanoparticles (FeONPs) and/or 30 mg of TiO_2_ nanoparticles (TiONPs) before pouring them into ~ 2.5 ml of 0.5M aqueous FeSO_4_ solution. The solution was then sonicated for around 45 min to disperse the nanoparticles in the CNT matrix. Following this, the sonicated material was spread on a cleaned glass slide before drying at ~60^o^C for 2 h. Thereafter, the resulting materials on the glass slide were coated with Mg using a thermal evaporator to deposit Magnesium film on the exposed side of the CNT-bots. This procedure imparted ‘Janus’ nature to the CNT-bots fabricated. Then, the thin layer was broken into tiny particles with the help of a sharp scrapper and stored for the use as CNT-bots. The presence of FeONPs ensured that the motor could undergo magnetotaxis inside any fluidic medium in presence of a magnet while the presence of Fe^2+^ ensured the motor could undergo chemotaxis inside a peroxide medium through the ejection of O_2_ bubbles. The coating of Mg facilitated decomposition of acidic-water and stimulated an acid-taxis through the ejection of H_2_ bubbles. Further, the presence of –COOH functionalization on the surface empower the same CNT-bot to eject carbon-dioxide (CO_2_) bubbles in the alkaline-water to stimulate alkali-taxis. Interestingly, the CNT-bot could also show directional acid- and alkali-taxes inside aqueous and peroxide mediums when a pH gradient was established. Further, doping of CNTs with TiONPs enabled phototaxy of the CNT-bots through photo-Fenton reaction. It may be noted here that the steps involved in the synthesis of CNT-bots were simple industrial unit operations which include agitation, mixing, spreading, drying, evaporation, coating, and scrapping. Thus, the CNT-bot fabrication could be scalable for future industrial applications.

**S2: Gas Chromatography**

**
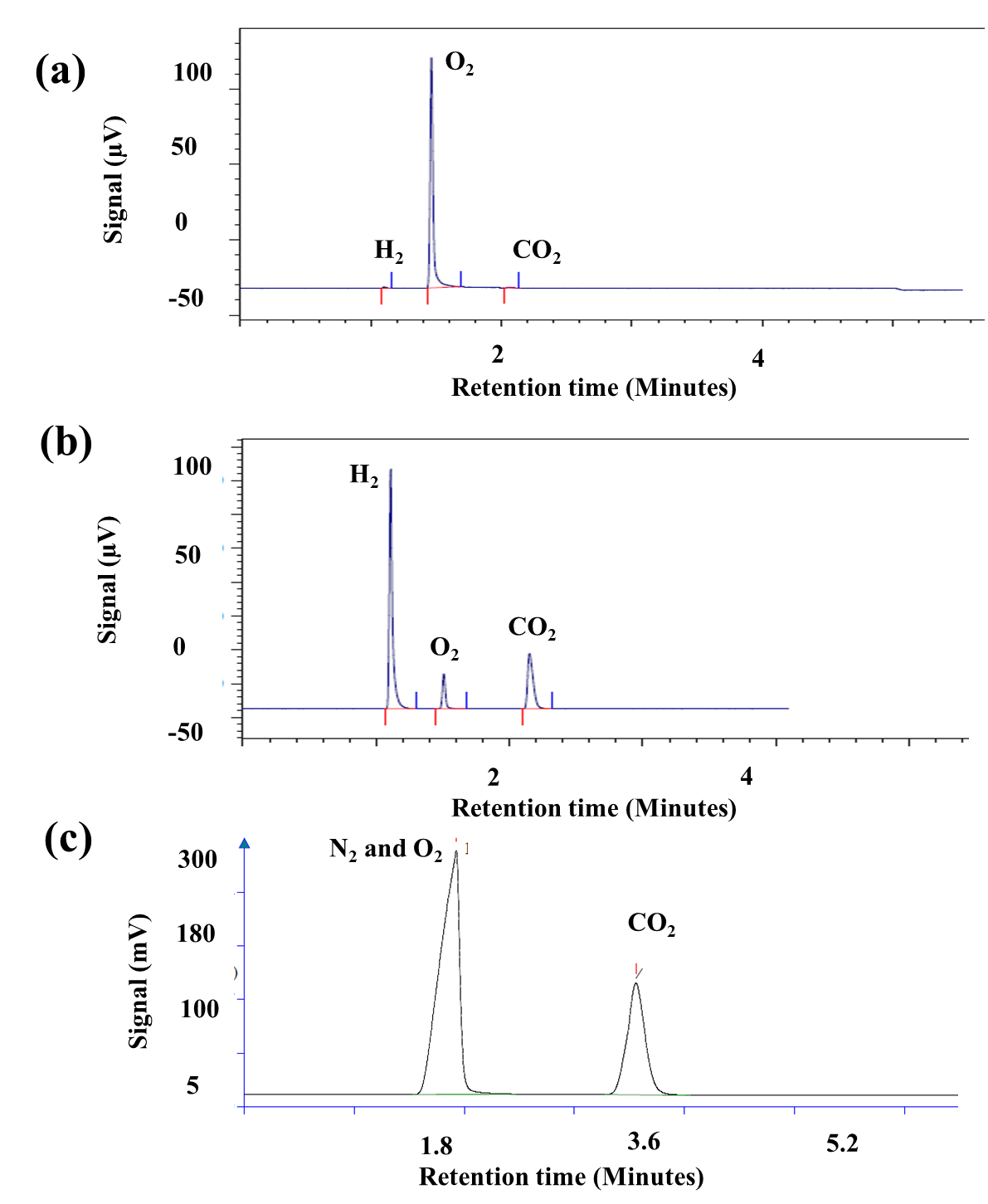
**

**Figure S2.** Plots (a) – (c) show the gas-chromatographs (GC) of the gases issued out when the CNT-bot reacted with 5% aqueous hydrogen peroxide, 0.05M aqueous HCl, and 0.5M aqueous sodium bicarbonate, respectively.

The gas chromatography (GC) characterization was done to confirm the gases issued out during the bubble propulsion of the CNT-bots. **Figures S2(a) – S2(c)** show the GC of the gases issued out when the CNT-bot reacted with 5% aqueous hydrogen peroxide, 0.05M aqueous HCl, and 0.5M aqueous sodium bicarbonate, respectively. For these experiments, in three separate stoppered culture tubes, we initially mixed 5 ml of respective fuels and 5 mg of the CNT-bots before the culture tubes were sealed. Following this, the gas accumulated at the empty space of the tube was withdrawn with the help of a GC syringe before injecting the same into the GC column. In the plot S1(a), the most intense peak was obtained for the oxygen gas, which was generated due to the Fenton reaction of CNT-bot with peroxide fuel. The presence of a little amount of hydrogen is also indicated in the same plot. This is because, as the hydrogen peroxide decomposed into water, the magnesium coating of the CNT-bot reacted with water to produce hydrogen. The peak of nitrogen also appeared because we were unable to remove air from the culture tube. The plot S1(b) shows an intense peak of hydrogen when CNT-bots reacted with 0.05 (M) HCl. In this case, the magnesium layer deposited on the CNT-bot reacted with acidic water to produce hydrogen. The plot S1(c) shows the peaks of oxygen, nitrogen, and carbon dioxide in which the intensity was high for the carbon dioxide owing to the reaction of carboxylic proton of the -COOH functionalized CNT-bots with sodium bicarbonate.

**S3: Chemical Kinetics**

**S3.1: Peroxide Decomposition by Fe^2+^ in CNT-bot**


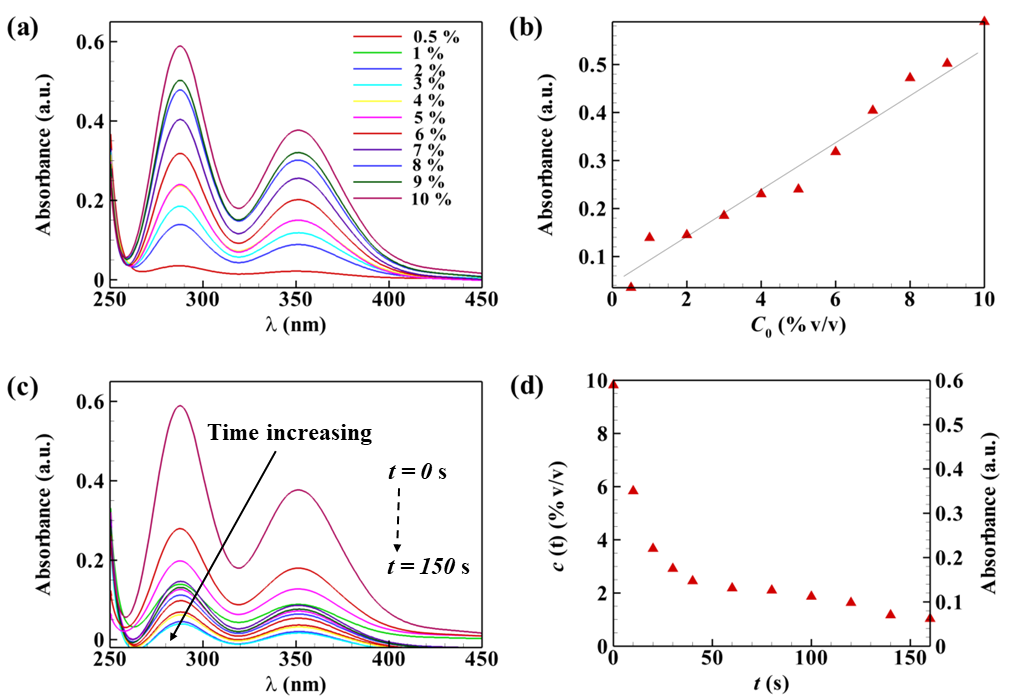


**Figure S3.** Image (a) shows the UV-Vis spectra of different known iodine solutions in the presence of excess iodide. Image (b) shows calibration curve, absorbance vs. peroxide concentration (*C*_0_) for peroxide concentration ranging from 1% (v/v) to 10% (v/v) aqueous peroxide solutions. Plot (c) shows *C*_0_ vs. time (*t*) plot for reaction between ferrous sulphate present in CNT bot and hydrogen peroxide solution.

The study of the chemical kinetics of the reaction between ferrous ion and hydrogen peroxide was studied iodometrically and with the help of UV spectrophotometry. It is well known that hydrogen peroxide reacts with iodide to give iodine, which can take up excess iodide ions to form triiodide in water. Thus, at the initial stage, known concentrations of aqueous peroxide solutions ranging from 1% to 10% (v/v) were prepared. Following this, we added 1:4 sulphuric acid to each peroxide solution before a stoichiometric excess of potassium iodide was added into the solutions, to facilitate the reaction, H_2_O_2_ + H_2_SO_4_ + 2KI → K_2_SO_4_ + I_2_ + 2H_2_O. Thus, addition of potassium iodide in the solution of hydrogen peroxide and potassium iodide liberated iodine. The iodine concentrations were observed to be higher for higher concentration of peroxide, as suggested by the stoichiometric equation. **Figure S3(a)** shows the absorption spectra of iodine at different peroxide concentrations. We observed two peaks of iodine at the wavelengths 280 nm and 360 nm, respectively. The calibration curve shown in the **Figure S3(b)** corresponds to the absorbance at 280 nm for different peroxide concentrations. Following this, we performed the same experiment with known volume and concentration of peroxide solution with known amount of particle loaded with known amount of ferrous sulphate. Once the reaction started, we collected equal amount of samples from the vessels at predetermined time intervals. The samples were diluted with the same ratio as it was done for making the calibration curve. **Figure S3(c)** shows the absorption vs. time plot for reaction between ferrous sulphate and hydrogen peroxide solution, which was obtained from the absorbance values at different time intervals and calibration curve.

**S3.2: Acidic Water Decomposition by Mg in CNT-bot**





**Figure S4.** The plot shows the variation in the concentration of hydrochloric acid in water (*C*_acid_) with time (*t*) during the reaction of aqueous acid solution with the CNT-bots.

The motion of the CNT-bots in water was due to bubble propulsion of hydrogen gas issuing out of the surface of the motor. In order to study the kinetics of this reaction, a 60 mg of CNT-bots were put in a bath of 0.01M HCl containing measured amount (50 ml) of HCl. The reactions started as soon as the CNT-bots were added and we took samples of 500 μl at every 60 s time interval. The concentration of the taken samples was measured by acid base titration method. Thus, an aliquot of 0.002M standard NaOH solution was prepared before the strength of the solution was measured against standard oxalic acid solution. All the samples withdrawn at different time intervals were diluted and titrated against standard 0.002M NaOH. From the titre values the concentration of acid was calculated at different time intervals. **Figure S4** shows the variation of (*C*_acid_) with time (*t*) due to the reaction, Mg + 2HCl = MgCl_2_ + H_2_.

**S4: Magnetic Hysteresis of CNT-bot**


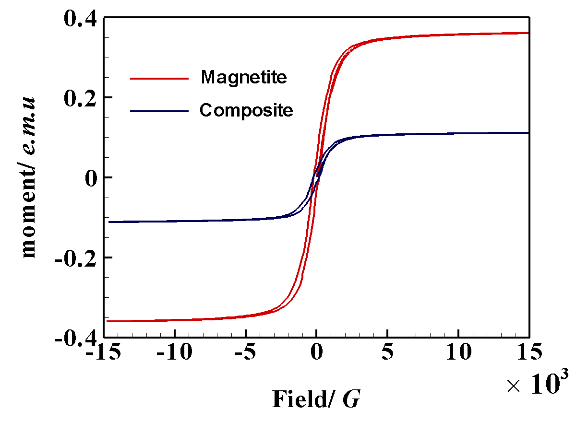


**Figure S5.** Shows magnetic hysteresis loops from vibrational scanning magnetometry (VSM) of pure magnetite (red line) and CNT-bots (blue line), respectively.

The ferromagnetic nature of the CNT-bots was characterised by vibrational scanning magnetometry (VSM). The CNT-bots were made magnetic by doping with magnetite nanoparticles with the MWCNTs in ~1:1 ratio. **Figure S5** shows the magnetic hysteresis loop of pure magnetite and CNT-bots. The minimum enclosed area of the loop indicates minimum magnetic loss and acceptable ferromagnetic nature of magnetite and CNT-bot particles. The plots also indicate that the dipole moment of the CNT-bots was nearly half of the pure magnetite owing to the non-magnetic MWCNTs.

**S5: Raman Spectra of Photo-CNT-bots**

**
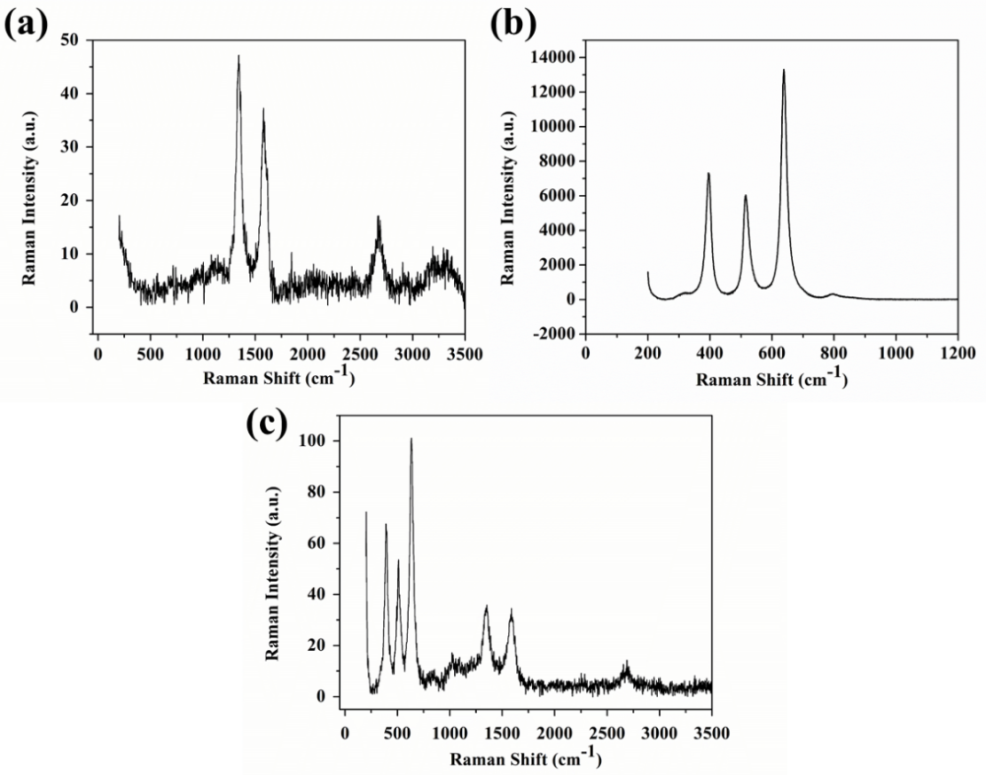
**

**Figure S6:** Plots (a) to (c) show Raman shifts of pristine MWCNT-COOH, TiONP, and the composite TiONP-FeSO_4_ doped MWCNT-COOH.

Photo-active CNT-bots were prepared by doping TiONPs and ferrous sulphate in the template of MWCNT. **Figure S6** shows Raman spectra of TiO_2_, MWCNT-COOH, and the composite material before magnesium deposition. **Figure S6(a)** shows the appearance of a prominent peak at 1323 cm^-1^ (D band), which is attributed to disorder in sp^2^ carbons available on the surface of MWCNTs. The peak at 1584 cm^-1^ (G band) is corresponding to the sp^2^ vibration of carbon atoms on the graphitic surface of MWCNT.[^2^](#_ENREF_2)^,^[^3^](#_ENREF_3) Similarly, **Figure S6(b)** show three distinct characteristic peaks of TiONPs at 380 cm^-1^, 447 cm^-1^ and 612 cm^-1^ indicating rutile structure of TiO_2_.[^4^](#_ENREF_4) **Figure S6(c)** shows that, in the composite material all the peaks were present, which also indicate a homogeneous mixture of MWCNT and TiONPs.

**S6: FESEM of Photo-CNT-bots**


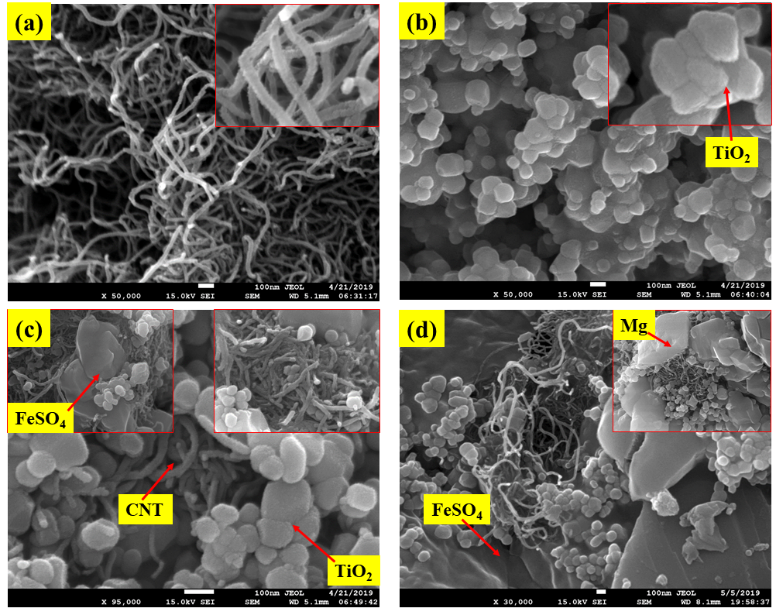


**Figure S7:** Images (a) and (d) show FESEM images of pristine –COOH substituted MWCNT, TiO_2_, TiO_2_ and ferrous sulphate doped MWCNT-COOH, and the ultimate photo-active CNT-bots after magnesium deposition.

The doped ferrous ions on the photo-active CNT-bot was the major reason behind the motion of the particles inside H_2_O_2_. FESEM images **S7(a) – (d)** show COOH substituted MWCNTs, TiONPs, Fe^2+^ and TiONP doped MWCNTs, and photo-active CNT-bots after magnesium deposition. In **Figure S7(c)** presence of all the materials such as MWCNT-COOH, TiO_2_, and ferrous sulphate crystals were observed in the composite material. **Figure S7 (d)** shows the presence of crystals of magnesium in the CNT-bots.

**S7: Control Experiments**





**Figure S8:** The plot shows the speeds of different CNT-bots in various mediums for the cases described in the legend.

In order to ascertain that the diverse locomotion shown in the manuscript were indeed due to the chemical and photonic triggers, we performed some control experiments, as shown in the **Figure S8**. The cases 1 to 4 show marginal or no motion for the CNT-bots under various controlled conditions. For example, in the cases of 1 to 2, absence of magnesium coating eliminated the possibility of hydrogen propulsions. In addition, cases 3 and 4 show marginal migration under UV light in absence of photo Fenton reaction. A higher magnitude of speed (~ 500 μm/s) in the case 5 (CNT-bot doped with Fe^2+^) showed the contribution of Fenton reaction. While the cases 6 (under magnetic field) and 7 (under UV light intensity) showed the external fields accelerated the bots to acquire high speeds of ~1500 to 2500 μm/s. Conclusively, this control studies proved that the contributions of Marangoni, thermophoretic, and diffusiophoretic forces were rather negligible.

**S8: Batch Dependency**


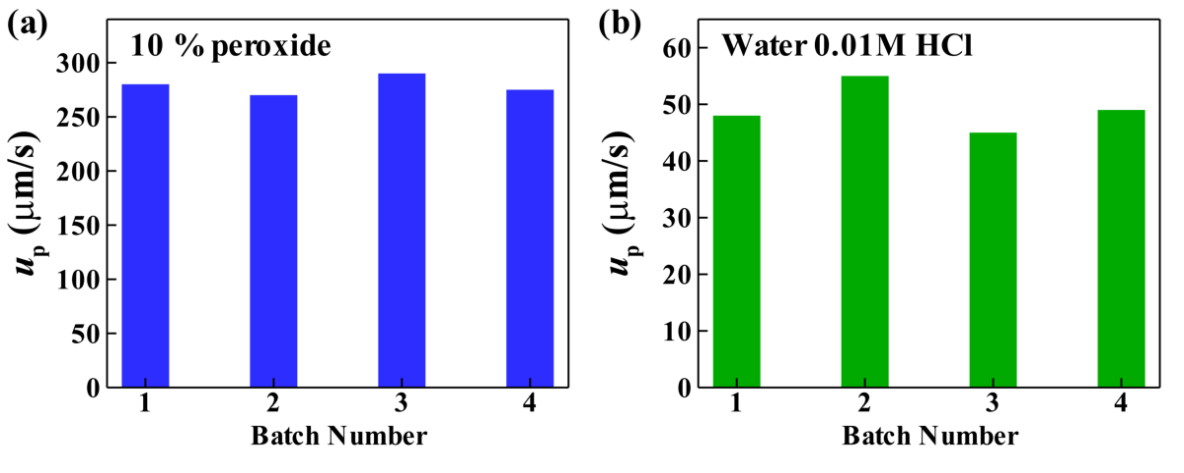


**Figure S9:** Plots (a) and (b) show a variation of CNT-bot speed for four different batches of motor fabrication in 10% hydrogen peroxide and acidic water (0.01M HCl), respectively.

In this section, we study the speeds of the CNT-bots for different batches of micromotor synthesized. All the steps of CNT-bot fabrication were followed systematically for each batch in order to remove batch dependency. **Figures S9(a) and S9(b)** show CNT-bot speeds for four different batches when placed in 10% hydrogen peroxide and acidic water (0.01M HCl), respectively. The plot suggests that different batches did not affect the particle motion to a large extent and the variations were within ± 5%.

**S9: Role of Incessant Motion of CNT-bots in Efficiency of Fuel Cell**


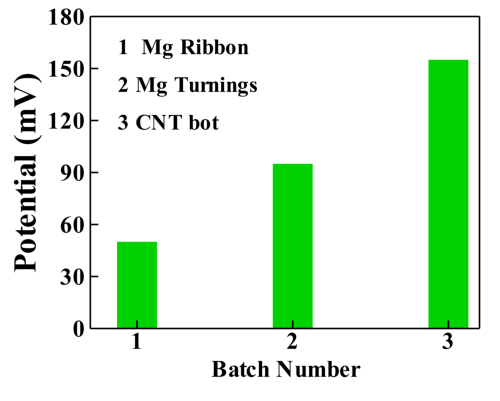


**Figure S10:** Shows maximum potential output across fuel cell end for three different feeds as hydrogen source.

In the **Supporting Video 11**, we showed fluctuating motions of the CNT-bots in peroxide and acidic water fuel medium. Movement of the CNT-bots facilitated the degassing and ejection of bubbles from fluid by incapacitating the hydrodynamic resistive force. At the same time, the generation rate of gases was also very high because of large surface to volume ratio of the particles. Hence, an enhanced gas generation rate, as a result of all the factors, made the fuel cell more efficient, as compared to direct feed of source materials for gas generation. **Figure S10** compares the output potential of the fuel cell for three different sources of hydrogen gas involving Mg ribbon (~50 mV), magnesium turnings (~95 mV) and CNT-bots (~ 155 mV). For all these experiments, we took 1 mg of magnesium in a reservoir containing 5 mL of 0.1 M of acidic water.

**S10. Electrochemical Study:**

A carbon tape was pasted on an ITO coated glass (1 cm × 2 cm) covering around 80% of the surface area while the rest 20% was kept vacant for connection purpose. Thereafter, the CNT-bots were dispersed on the carbon tape due exploiting the presence of the adhesive layer. A cyclic voltammetry (CV) study of this working electrode was made by using ~10% H_2_O_2_ as electrolyte, Ag/AgCl electrode as reference electrode, and Pt as counter electrode from -3 V to +3 V with a scan rate of 50 mV/s. The experiments were also performed with the working electrodes of carbon tape without the CNT-bots as a control. The **Figure S11** clearly shows ~ 1.5-fold increase of current in the case of electrode fabricated with CNT-bots. The reaction of the CNT-bots with ~ 10% H_2_O_2_ generated excess electrons near the electrode surface and caused an enhancement of current passing through the counter electrode.

**

**

**Figure S11:** Shows the cyclic voltammetry plots of an electrochemical cell having ~ 10% H_2_O_2_ taking Ag/AgCl and Pt as the reference and counter electrodes while the CNT-bots on carbon tape as the working electrode. The black curve indicates CV of carbon tape electrode without CNT-bots and red curve signifies CV with carbon tape electrode with CNT-bots.

**S11: EDX Spectroscopy**


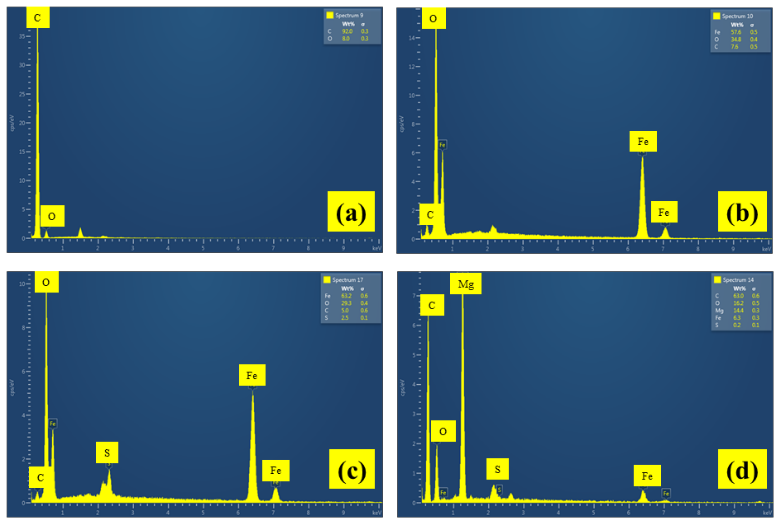


**Figure S12.** Images (a) – (d) show the EDXS spectra of pristine carboxylated MWCNT, magnetite doped MWCNTs, ferrous sulphate treated and magnetite doped MWCNTs, and CNT-bots after final magnesium coating, respectively.

Presence of different elements in the CNT-bots was confirmed by Electron Dispersive X-ray Spectroscopy (EDXS). **Figures S12(a) – S12(d)** show the EDXS spectra of pristine carboxylated MWCNT, magnetite doped MWCNTs, ferrous sulphate treated and magnetite doped MWCNTs, and CNT-bots after final magnesium coating, respectively. The plot (a) shows the peaks of carbon and oxygen. The plots (b) and (c) show the peaks of carbon, oxygen, iron and sulphur, which indicated magnetite and ferrous sulphate doping. Plot (d) shows the presence of all carbon, oxygen, iron, sulphur, and magnesium on the CNT-bot.

## **S12: XRD Analysis**

The XRD of the pristine magnetite and MWCNT and subsequent composites after doping and depositions were also performed. **Figure S13(a)** shows the XRD pattern of pristine magnetite nanoparticles with the characteristic peaks at, 220, 311, 400, 442, and 511 for magnetite. **Figure S13(b)** shows the XRD spectra of magnetite doped and ferrous sulphate treated MWCNTs before deposition of magnesium. In this case, both the peaks for magnetite and ferrous sulphate were observed at 220, 311, 400, 442, and 511 for magnetite^1^ and ferrous sulphate[^1^](#_ENREF_1) at 110, 020, 112, 130,310, 222, and 242.


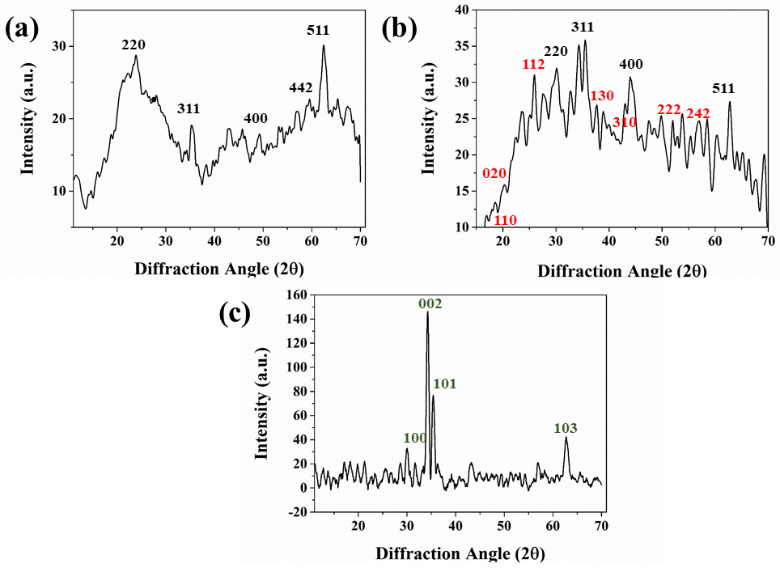


**Figure S13.** Image (a) shows XRD spectra of pristine magnetite nanoparticles. Image (b) shows magnetite doped and ferrous sulphate treated MWCNTs. Image (c) shows XRD spectra of the CNT-bots after the deposition of magnesium layer.

The **Figure S10(c)** shows the XRD spectra of the CNT-bot after the addition of the layer. In addition to the previous peaks, we observed the presence of peaks at, 100, 002, 101, and 103, corresponding to magnesium.^3^

**S13: Description of Videos**

**Supporting Video 1:** The video shows the details of the random chemotaxis of the CNT-bot in the 10% (v/v) aqueous peroxide medium, as depicted by the snapshots in the Figure 2(a) of the main manuscript. The video corresponds to the oxygen bubble propulsion of the CNT-bot.

**Supporting Video 2:** The video shows the details of the random chemotaxis of the CNT-bot in the aqueous medium, as depicted by the snapshots in the image set Figure 2(b) of the main manuscript. The video corresponds to the hydrogen bubble propulsion of the CNT-bot.

**Supporting Video 3:** The video shows the details of the random chemotaxis of the CNT-bot in the acidic water medium, as depicted by the snapshots in the image set Figure 2(c) of the main manuscript.

**Supporting Video 4:** The video shows the details of the random chemotaxis of the CNT-bot in the aqueous sodium bicarbonate solution medium, as depicted by the snapshots in the image set Figure 2(d) of the main manuscript.

**Supporting Video 5:** The video shows the details of the directed chemotaxis of the CNT-bot, as depicted by the snapshots in the image set Figure 2(e) of the main manuscript. The video shows the directional acid-taxis of the motor in water when acid was dripped through a thread inside the water bath, as shown.

**Supporting Video 6:** The video shows the details of the directed chemotaxis of the CNT-bot, as depicted by the snapshots in the image set Figure 2(f) of the main manuscript. The video shows the directional alkali-taxis of the motor in water when alkali was dripped through a thread inside the peroxide bath, as shown.

**Supporting Video 7:** The video shows the details of the directed chemotaxis of the CNT-bot, as depicted by the snapshots in the image set Figure 2(g) of the main manuscript. The video shows the directional alkali taxis of the motor in water when sodium bicarbonate was dripped through a thread inside the water bath, as shown.

**Supporting Video 8:** The video shows the details of the magnetotaxis of the CNT-bot, as depicted by the snapshots in the image set Figure 2(h) of the main manuscript. The video shows the motion in water when the magnetic field strength was 155 Gauss.

**Supporting Video 9:** The video shows the details of the phototaxy of the CNT-bot, as depicted by the snapshots in the image set Figure 2(i) of the main manuscript. It shows the directed motion of the photo-active CNT-bot under the guidance of UV light source.

**Supporting Video 10:** The video shows the power generation in a PEM Fuel cell, as described in theFigure 4 of the main manuscript.

**Supporting Video 11**: The video shows the movement of the motors leading to generation and degassing of the fuels suitable for power generation through the PEM fuel cell.

**Supporting Video 12**: The video shows the decolouration of the methylene blue solution with time when the CNT-bots were added, as described in the Figure 5 of the main manuscript.

**References:**

1. Zhang, L. et al. Facile synthesis of iron oxides/reduced graphene oxide composites: application for electromagnetic wave absorption at high temperature. *Sci. Rep.* **5**, 9298 (2015).

2. Abdulla, S., Mathew, T.L. & Pullithadathil, B. Highly sensitive, room temperature gas sensor based on polyaniline-multiwalled carbon nanotubes (PANI/MWCNTs) nanocomposite for trace-level ammonia detection. *Sens. Actuators, B* **221**, 1523-1534 (2015).

3. Machado, F.M., Bergmann, C.P., Lima, E.C., Adebayo, M.A. & Fagan, S.B. Adsorption of a textile dye from aqueous solutions by carbon nanotubes. *Mater. Res.* **17**, 153-160 (2014).

4. Ohsaka, T., Izumi, F. & Fujiki, Y. Raman spectrum of anatase, TiO2. *J. Raman Spectrosc.* **7**, 321-324 (1978).
